# Role of long non-coding RNAs in glucose metabolism in cancer

**DOI:** 10.1186/s12943-017-0699-3

**Published:** 2017-07-24

**Authors:** Chunmei Fan, Yanyan Tang, Jinpeng Wang, Fang Xiong, Can Guo, Yumin Wang, Shanshan Zhang, Zhaojian Gong, Fang Wei, Liting Yang, Yi He, Ming Zhou, Xiaoling Li, Guiyuan Li, Wei Xiong, Zhaoyang Zeng

**Affiliations:** 1 The Key Laboratory of Carcinogenesis of the Chinese Ministry of Health, Xiangya Hospital, Central South University, Changsha, Hunan China; 2 The Key Laboratory of Carcinogenesis and Cancer Invasion of the Chinese Ministry of Education, Cancer Research Institute, Central South University, Changsha, Hunan China; 3 Hunan Key Laboratory of Nonresolving Inflammation and Cancer, Disease Genome Research Center, The Third Xiangya Hospital, Central South University, Changsha, Hunan China

**Keywords:** LncRNAs, Glucose metabolism, Warburg effect, Signaling pathway, Targeted therapy

## Abstract

Long-noncoding RNAs (lncRNAs) are a group of transcripts that are longer than 200 nucleotides and do not code for proteins. However, this class of RNAs plays pivotal regulatory roles. The mechanism of their action is highly complex. Mounting evidence shows that lncRNAs can regulate cancer onset and progression in a variety of ways. They can not only regulate cancer cell proliferation, differentiation, invasion and metastasis, but can also regulate glucose metabolism in cancer cells through different ways, such as by directly regulating the glycolytic enzymes and glucose transporters (GLUTs), or indirectly modulating the signaling pathways. In this review, we summarized the role of lncRNAs in regulating glucose metabolism in cancer, which will help understand better the pathogenesis of malignant tumors. The understanding of the role of lncRNAs in glucose metabolism may help provide new therapeutic targets and novel diagnostic and prognosis markers for human cancer.

## Background

Metabolism is one of the basic attributes of life. In the 1920s, Warburg found that tumor cells exhibit a special metabolic phenotype. One of the features of this phenotype is that despite adequate availability of oxygen, cancer cells still tend to generate energy from glycolysis, rather than depending on oxidative phosphorylation, which produces more ATP per molecule of glucose. This phenomenon is known as the “Aerobic glycolysis” or “Warburg effect” [1, 2]. It often results in increased glucose uptake and accumulation of ATP and lactic in the cancer cells.

**Table 1:** LncRNAs and their targets in the regulation of glucose metabolism in cancer

Items	Targets	LncRNAs	Tumor types	References
GLUTs	GLUT1	LncRNA NBR2	Kidney cancer	[32]
	GLUT4	LncRNA CRNDE	Colorectal neopasia	[33]
Enzymes	HK2	LncRNA PVT1	Osteosarcoma	[34]
	PKM2	LncRNA H19	Liver cancer	[36]
	G6P, PEPCK	LncRNA GAS5	Cervical/Hepatocellular cancer	[37]
	Pyruvate carboxylase	LncRNA GCASPC	Gallbladder cancer	[39]
	PFKFB2	LINC00092	Ovarian cancer	[40]
Oncogenes	c-Myc	PCGEM1, LncRNA-MIF	various cancer	[42, 43]
HIF	HIF and VHL	LincRNA p21, MALAT1	Hepatocellular/Breast cancer et al	[50, 51]
	HIF-1α	LncRNA-LET, H19, LINK-A	Breast cancer	[52–54]
	miR-145 and HIF-1α	Linc-ROR	Hepatocellular cancer	[55]
PI3K/AKT	PTEN	PTENpg1, HOTAIR	Prostate/Tongue squamous carcinoma	[65, 66]
	Akt	ANRIL	Nasopharyngeal carcinoma	[67]
	Let-7	H19	-	[69]
AMPK	LKB1	LINC00473	Lung cancer	[74]
	AMPK	LncRNA NBR2	various cancer	[77]
Wnt/Snail	EMT	LncRNA CTD903, UCA1	Colorectal cancer, breast cancer	[80, 81]
STAT	STAT1 and PolyII	NRCP	Ovarian cancer	[82]
	STAT3	UCA1	Bladder cancer	[86]
p53	Mutant p53, PKM2	LncRNA CUDR	Hepatocarcinogenesis	[94]
	p53 protein	MEG3, Wrap53	various cancer	[95, 97]
	p53, hnRNP-K	LincRNA p21, MALAT1	various cancer	[49, 98]
	p53	Linc-ROR	various cancer	[49]

Initially, Warburg speculated that the mitochondrial function in tumor cells might be impaired, making it obligatory for the tumor cells to depend on aerobic glycolysis [3]. But later work found that mitochondrial function is not damaged in most tumor cell types [4]. Further studies have shown that proliferating cells require not only ATP, but also nucleic acids, fatty acids, proteins, and membrane phospholipids. Glycolysis can provide substrates and intermediates required for the synthesis of the aforementioned biological macromolecules. Glycolysis generates small molecule precursors or intermediates that contribute to cell proliferation, such as acetyl-CoA, intermediates of non-essential amino acids, and ribose for nucleotide synthesis to meet the needs of rapid DNA replication [3, 5]. Glycolysis produces lower quantities of reactive oxygen species (ROS). ROS can induce apoptosis or senescence in tumor cells under oxygen stress [6]. Since mitochondrial oxidative phosphorylation produces higher levels of ROS, it is advantageous for the tumor cells to depend on glycolysis for their energy needs. Although glycolysis produces less ATP than oxidative phosphorylation, glycolytic intermediates provide the carbon sources that are required for rapid cell proliferation [7]. The lactate generated by glycolysis lowers the pH of the extracellular matrix (ECM) [8]. Acidic microenvironment promotes tumor invasion and metastasis and confers resistance to radiation therapy [9, 10]. Thus, the Warburg effect is an optimized way that tumor cells harness cellular stress to thrive. It also suggests that cancer is a metabolic disease. The most direct way of altering glucose metabolism is by affecting the metabolic enzymes or kinases. However, some signaling pathways also play important roles in glucose metabolism. Regulation of enzymes, kinases and signaling pathways may directly or indirectly affect glucose metabolism in cancer. Changes at mRNA and protein levels have been shown to be involved in reprogramming the glucose metabolism in tumor cells [11, 12].

A very large part of the more than 3 billion base pair long human genome is transcribed, but less than 2% of the genome encodes proteins. Most of the transcripts are not translated into proteins. These are referred to as non-coding RNAs (ncRNAs), which are longer than 200 nucleotides (NT), are called long non-coding RNAs (lncRNAs) [13–20]. LncRNAs are involved in a variety of important regulatory processes, at the transcriptional and post-transcriptional levels [21–27], and in epigenetic modifications [28–31] that play complex and precise regulatory roles in development and gene expression. LncRNAs can also regulate glucose metabolism in tumor cells [32–35]. The regulatory mechanism of lncRNAs is extremely complicated and merits systematic and in-depth research. A large number of studies have shown that lncRNAs can affect genes involved in glucose metabolism [36]. Therefore, we focused on the ways and mechanisms by which lncRNAs regulate glucose metabolism in cancer, which may help advance the understanding the complex regulatory network of cancer metabolism and provide a better theoretical basis for clinical diagnosis and treatment. LncRNAs and their targets in the regulation of glucose metabolism in cancer are summarized in Table 1.

## LncRNAs regulate enzymes, regulatory molecules, and oncogenes involved in glucose metabolism in cancer

### LncRNAs regulate glucose uptake via altering the expression of glucose transporters

Glucose transporters (GLUTs) are membrane proteins that transport glucose from the capillaries into cells and play an important role in cellular glucose metabolism. So far, 13 members of the GLUT family have been identified, out of which GLUT1, GLUT3, and GLUT4 are closely involved in glucose metabolism in cancer. Under normal physiological conditions, GLUTs transport glucose rapidly. GLUTs are often upregulated in malignant tumor cells, expediting the glucose transport further.

**Fig. 1:**
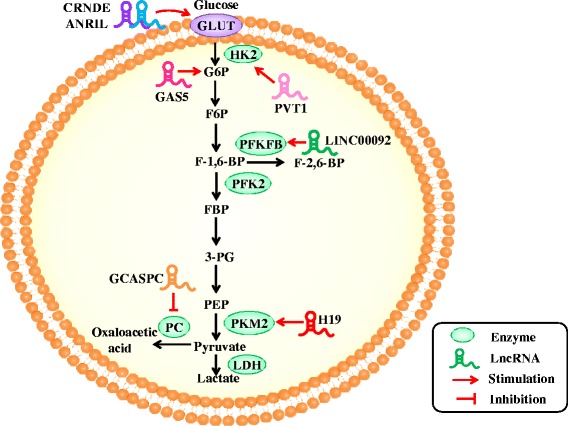
LncRNAs regulate the molecules involved in glucose metabolism in cancer. LncRNAs regulate glucose uptake and glycolytic flux by modulating GLUTs and glycolic enzymes

LncRNA NBR2 regulates AMPK activity and is induced by glucose starvation. However, Liu et al. showed that knocking out NBR2 does not affect phenformin-induced AMPK activity, but attenuates the expression of GLUT1, thereby reducing glucose uptake [37]. LncRNA Colorectal neoplasia differentially expressed (LncRNA-CRNDE) regulates gene expression by epigenetic modification. The intron region of this gene has a highly conserved sequence (gVC-In4). Ellis demonstrated that knocking out gVC-In4 in HT29 cells reduced the amount of lactic acid produced in cancer cells. They further showed that the reduction in lactic acid production was due to the decrease in the efficiency of aerobic glycolysis or conversion of pyruvate to acetyl-CoA. They also found that the expression of GLUT4 was reduced, indicating that CRNDE modulates the level of GLUT4 positively [38] (Fig. 1).

### LncRNAs influence glycolysis by regulating enzymes or kinases

HK2 was a direct target of miR-497, long non-coding RNA PVT1 acts as molecular sponge to repress miR-497, as a result, PVT1 promotes glycolysis and cell proliferation in osteosarcoma and form a PVT1/miR-497 axis in the Warburg effect through regulation of HK2 expression [39] (Fig. 1).

Pyruvate kinase (PK) is the last rate-limiting enzyme in glycolysis. Allosteric as well as covalent modifications can affect PK activity. Four isoenzymes of PK have been identified so far: M, K, L, and R types. The aberrant expression of pyruvate kinase M2 (PKM2) is most common in tumor cells [40]. PKM2 determines the proportion of carbons derived from glucose that are used for glycolytic energy production [41]. In the breast cancer cell line MCF-7, the cytoplasmic promyelocytic leukemia tumor suppressor protein (PML-TSP) interacts directly with PKM2. Overexpression of a mutated form of PML-TSP, which was generated by mutagenesis of the nuclear localization signals of PML-TSP, suppressed PKM2 activity and accumulation of lactate [42]. Li et al. illustrated that miR675 inhibits the expression of heterochromatin protein 1α (HP1α), leading to changes in histones. miR675 also upregulates lncRNA H19 via EGR1 activation. H19 can induce and activate PKM2, which is essential for Waburg effect and tumorigenesis in liver cancer [43] (Fig. 1).

LncRNA GAS5 binds to the DNA binding domain of the adrenocorticotropic hormone receptor, thereby preventing its binding to the regulatory region of the gene. GAS5 inhibits the expression of 6-phosphoglucanase (G6Pase) and phosphoenolpyruvate carboxykinase (PEPCK) [44], enzymes that play key roles in glucose metabolism, thereby inhibiting gluconeogenesis and glycogenolysis [45]. Thus, the role of GAS5 in glucose metabolism is undoubtedly of great significance (Fig. 1).

Pyruvate carboxylase (PC), an enzyme that convert pyruvate to oxaloacetate, has been proved to play an important role in cancer cell metabolism and proliferation. In gallbladder cancer, GCASPC binds to pyruvate carboxylase, reduces its level and activity by promoting the instability of PC, thereby inhibiting cell proliferation [46] (Fig. 1).

LINC00092 is upregulated in ovarian cancer. It inhibits one of the glycolytic enzymes, fructose-2,6-bisphosphatase (PFKFB2), thereby altering glycolysis, which in turn promotes metastasis and sustains the local supportive function of cancer-associated fibroblasts (CAFs) [47–50] (Fig. 1). Although many enzymes involved in glucose metabolism have been described, there are few reports that discuss how lncRNAs affect the levels of metabolism by influencing these enzymes. It is also necessary to investigate whether lncRNAs are associated with other enzymes involved in glucose metabolism.

### LncRNAs affect glycolysis by regulating oncogenes

**Fig. 2:**
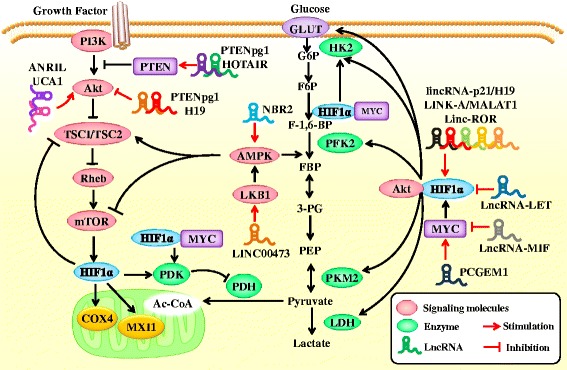
Role of lncRNA-mediated HIF, PI3K/AKT/mTOR and LKB1-AMPK pathways in glucose metabolism in tumor cells. LncRNAs can regulate HIF-1α protein synthesis and stability, thus modulating HIF-1-mediated metabolic reprogramming. The rate of translation of HIF-1a mRNA in cancer cells is dependent upon the activity of the mammalian target of rapamycin (mTOR), which in turn is determined by the activity of upstream tumor suppressor proteins and oncoproteins. HIF-1α plays a key role in stimulating glycolic enzymes and in blocking mitochondrial activity. LncRNAs can also regulate Akt and AMPK pathways. Akt may increase oxidative phosphorylation by enhancing metabolic coupling between glycolysis and oxidative phosphorylation, through facilitating the association of mitochondrial hexokinase with VDAC and mitochondria. Akt enhances glycolytic flux via multiple mechanisms. First, it increases glucose uptake and flux. Second, hyperactive Akt activates mTORC1, which promotes HIF1α accumulation under normoxic conditions and increases GLUT1, HKII, and lactate dehydrogenase (LDH) levels. Finally, Akt-increased cellular ATP levels serve to maintain low AMPK activity, which is required for full activation of mTORC1

**Fig. 3:**
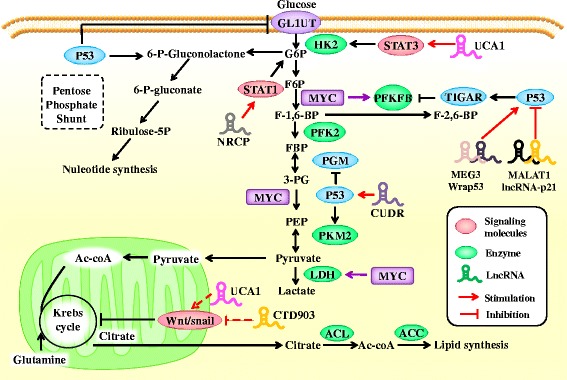
Role of lncRNA–mediated Wnt/Snail, STAT and p53 pathways in glucose metabolism in tumor cells. LncRNA can modulate the expression of Wnt/Snail, STAT and p53 expression and exert regulatory effect on glucose metabolism. p53 plays a key role in the process of glycolysis and oxidative phosphorylation, through interacting with various molecules or enzymes, such as TIGAR, GLUTs and PGM, thus affecting several key biological processes including glucose uptake and pyruvate conversion. LncRNAs can affect expression of glycolic enzymes through STAT pathways and modulate mitochondrial activity via Wnt/Snail

Accumulating evidence shows that *MYC* oncogene dysregulation is a common event in tumorigenesis. *MYC* oncogene encodes the transcription factor, c-Myc, which promotes cell growth and proliferation. Jung-whan Kim demonstrated that hypoxia-inducible factor 1 (HIF-1) cooperates with dysregulated c-Myc to promote glycolysis by inducing hexokinase 2, which catalyzes the first step of glycolysis, and pyruvate dehydrogenase kinase 1, which inactivates pyruvate dehydrogenase and diminishes mitochondrial respiration [51]. The prostate cancer marker, lncRNA PCGEM1, can influence a variety of metabolic pathways such as glucose metabolism, PPP, nucleic acid and fatty acid biosynthesis, and tricarboxylic acid cycle, at the transcriptional level. Significantly, PCGEM1 binds directly to the promoters of target genes, physically interacts with c-Myc, promotes chromatin recruitment of c-Myc, and enhances its transactivation activity [52]. Under normal oxygen conditions, c-Myc regulation of the glycolytic genes promotes glucose metabolism. The interaction of lncRNA with c-Myc inhibitory factor (MIF) reduces the level of c-Myc protein, thereby inhibiting glycolysis. Mechanistically, lncRNA-MIF acts as an endogenous competitive RNA for miR-586, reducing the inhibitory effect of miR-586 on Fbxw7, an E3 ubiquitin ligase that regulates c-Myc protein stability. Thus, lncRNA-MIF increases the expression of Fbxw7 and reduces the c-Myc protein level. There is a feedback loop between c-Myc and lncRNA-MIF, which regulates the expression of c-Myc protein and glucose metabolism [53] (Figs. 2 & 3).

## LncRNAs affect glucose metabolism by regulating metabolism-related signaling pathways

### HIF signaling pathway

HIF is a nuclear transcription factor that is produced by cancer cells adapting to hypoxic environments [54]. Activation of HIF-1α contributes to Warburg effect, partly through the upregulation of GLUTs, thereby increasing glucose uptake [55] or by increasing the expression of glycolytic enzymes [56, 57] or by inhibiting oxidative phosphorylation [58]. These studies indicate that the Warburg effect is not caused just by hypoxia, but rather through a more specific regulation of transcription, in which HIF-1 increases the expression of most glycolytic enzymes.

Hypoxia is thought to be related to Warburg effect, although the underlying mechanism is not yet clear. LincRNA-p21 was originally thought to be a p53-induced lncRNA that regulated P53-triggered apoptosis in murine models [59]. However, it is not associated with apoptosis in human tissues. LincRNA-p21 is a hypoxia-responsive lincRNA that competes with HIF-1α to bind to the von Hippel-Lindau tumor suppressor protein (pVHL) and prevents the formation of HIF-1α-pVHL, thus inhibiting the ubiquitinated degradation of HIF-1α. pVHL is a component of ubiquitin ligase complex that binds to HIF-1α and routes it to the proteasome degradation pathway. Thus, lincRNA-p21 plays an important role in hypoxia-induced glycolysis. Under hypoxic conditions, HIF-1α-induced lincRNA-p21 stabilizes HIF-1α, forming a positive feedback loop. But this loop is not always activated because hypoxic stimulation may slow down [60]. In human hepatic epithelial cells (L-02), arsenite increases the expression of glycolysis-related genes, including HK2, Eno-1, and Glut-4. In L-02 cells exposed to arsenite, the lncRNA, metastasis-associated lung adenocarcinoma transcript 1 (MALAT1), and HIF-α, are overexpressed. Moreover, MALAT1 enhances arsenite-induced glycolysis by promoting the disassociation of HIF-1α from VHL, preventing VHL-mediated ubiquitination of HIF-1α, which causes the accumulation of HIF-1α [61]. However, the overexpression of lncRNA-LET results in a decrease in the expression of HIF-1α [62]. Hypoxia also induces LncRNA H19, which is involved in hypoxia-induced signal transduction processes in cancer cells, thereby altering glucose metabolism [63]. Lin reported that an lncRNA in cytoplasm, long intergenic non-coding RNA for kinase activation (LINK-A), is involved in the metabolic reprogramming in triple-negative breast cancer [64]. LINK-A facilitates the recruitment of BRK to the EGFR-GPNMB complex and activates BRK kinase. The BRK-dependent phosphorylation of HIF1α at tyrosine 565 interferes with hydroxylation of proline 564, thereby stabilizing HIF1α. LINK-A promotes the metabolic reprogramming and tumor progression in triple negative breast cancer by activating HIF1α. Takahashi et al. reported that linc-ROR is associated with hypoxia response and can act as a molecular sponge of miR-145 to regulate HIF-1α and its target genes such as VEGF, TGF-β, and PDK1 [65] (Fig. 2).

### PI3K/AKT/mTOR signaling pathway

Phosphoinositide 3-kinase (PI3K) signaling pathway is involved in glucose metabolism even in insulin-free tissues. PI3K indirectly increases the expression of GLUTs and enzymes by modulating Akt and mammalian target of rapamycin (mTOR). Akt-related metabolic factors include apoptosis-related kinases and GLUTs. Activation of Akt can increase cellular ATP production and oxygen consumption [66, 67]. In short, Akt plays a pivotal role in determining the pathway of ATP production; glycolysis or oxidative phosphorylation. Akt regulates glycolysis via multiple mechanisms: (1) increasing the expression of GLUTs [68]; (2) enhancing the expression of glycolytic enzymes such as HK2, PKM2 [67, 69, 70] or inhibiting mitochondrial oxidative phosphorylation [71, 72]; (3) activating mTORC1, which in turn increases HIF-1 levels [73, 74].

Polisenno found that PTEN pseudogene (PTENpg1) can regulate the expression level of *PTEN*, and inhibit tumor growth by inhibiting Akt signaling pathway [75]. The PTENpg1 and *PTEN* 3′ UTR contain a highly conserved domain, and a non-conserved domain. PTENpg1 can protect *PTEN* mRNA by blocking the interaction of miRNA and *PTEN* in the form of miRNA decoy. Similarly, hox transcript antisense RNA (HOTAIR) is overexpressed in a variety of tumors. HOTAIR in human tongue squamous cell carcinoma is associated with increased *PTEN* methylation. PTEN inhibits Akt signaling pathway and regulates glucose metabolism [76] (Fig. 2).

LncRNA ANRIL is upregulated in nasopharyngeal carcinoma. ANRIL increases the uptake and utilization of glucose in aerobic glycolysis by increasing the phosphorylation of Akt and activating the mTOR signaling pathway, resulting in the upregulation of GLUT1 [77]. Kallen noted that H19 harbors both canonical and non-canonical binding sites for the let-7 family of micro-RNAs, which plays important roles in development, cancer, and metabolism. LncRNA H19, acts as a molecular sponge to inhibit miRNA Let-7 activity [78]. H19 is highly expressed in a variety of human cancers. H19 expression is inhibited via PI3K/AKT-dependent phosphorylation of the miRNA processing factor KSRP. Inhibition of H19 expression increases let-7 levels, resulting in the impairment of insulin/PI3K/AKT pathway, leading to reduced glucose uptake [79] (Fig. 2).

### LKB1-AMPK signaling pathway

AMP activated protein kinase (AMPK) is a highly conserved cellular energy sensor that is necessary for glucose homeostasis [80, 81]. Activation of AMPK triggers the activation of TSC2 complex, leading to inactivation of mTOR-activated GTP-binding protein Rheb. mTOR also inhibits AMPK directly [56]. Under energy deficit conditions, AMPK enhances the activity of TSC2 by phosphorylating it and thus protects cells from apoptosis [82].

Liver kinase B1 (LKB1) is a threonine/serine kinase and tumor suppressor that regulates cell growth and energy metabolism by regulating the activity of m-TOR. Knocking down LKB1 promotes tumor cell proliferation, with increased uptake and utilization of glucose, enhanced ATP levels, and biosynthesis of macromolecules. In LKB1-deficient cells, this metabolic reprogramming process relies on HIF-1α, which exerts its antagonism by inhibiting m-TORCI [12, 83]. LINC00473 is a nuclear lncRNA that interacts with NONO, a component of the cAMP signaling pathway. LINC00473 is highly expressed in human non-small cell lung cancer and is associated with LKB1 dysregulation. LINC00473 was induced by LKB1 inactivation and subsequent cyclic AMP-responsive element-binding protein (CREB)/CREB-regulated transcription coactivator (CRTC) activation [84] (Fig. 2).

LncRNA NBR2 is induced by LKB1-AMPK signaling pathway under conditions of energy stress. NBR2 can act as tumor suppressor by enhancing the activity of AMPK kinase [85, 86]. LKB1 can activate AMPK, followed by AMPK phosphorylation. AMPK activates a series of downstream target genes, inhibiting ATP-depleted anabolism and activating ATP-induced catabolism. Glucose starvation can induce the phosphorylation of AMPK or acetyl-CoA carboxylase. Knocking down NBR2 significantly attenuates phosphorylation of AMPK and mTORC1 inactivation, suggesting the presence of a NBR2-AMPK feedback loop mechanism [87]. Adenosine kinase alleviates ATP depletion by converting two ADPs into one ATP and one AMP, which maintains the ATP/ADP ratio when ATP is rapidly decreasing. However, excessive accumulation of AMP activates LKB1-dependent AMPK, which in turn activates downstream target genes to replenish the energy currency of the cell [88] (Fig. 2).

### Wnt/snail signaling pathway

Su Yeon Lee et al. showed that Wnt inhibits mitochondrial respiration via inhibiting cytochrome c oxidase and promotes glycolysis by inducing pyruvate carboxylase, a key anaplerotic enzyme. This process relies on the β-catenin/T-cell factor 4/Snail signaling pathway. Knocking down E-cadherin repressed mitochondrial respiration and stimulated glycolysis via Snail activation, indicating that EMT may contribute to Wnt/Snail-mediated regulation of mitochondrial respiration and glucose metabolism [89].

In metastatic lung adenocarcinoma, lncRNA-CTD903 inhibited Wnt/β-catenin and subsequently inhibited the expression of transcription factors, Twist and Snail, to influence EMT and inhibit the invasion and metastasis of lung adenocarcinoma cells [90]. In the breast cancer cell line MDA-MB-231, lncRNA UCA1 contributes to the stimulation of EMT through Wnt/β-catenin signaling pathway, thus promoting the invasion and metastasis of breast cancer cells [91]. We speculate that lncRNA could indirectly alter glucose metabolism in cancer by affecting EMT via Wnt/Snail pathway (Fig. 3).

### STAT signaling pathway

LncRNA NRCP is upregulated in ovarian cancer and promotes tumor cell growth and proliferation by stimulating glycolysis. Rupaimoole demonstrated that NRCP promotes STAT1 binding to RNA polymerase II. When the expression of NRCP was silenced by the introduction of siRNA–NRCP into the tumor microenvironment, binding of RNA polymerase II to STAT1 decreased, indicating that NRCP acts as an intermediate in the binding of STAT1-RNA polymerase II. Further studies have shown that NRCP binds to STAT1 and RNA polymerase II, leading to an increase in the expression of downstream target genes such as glucose-6-phosphate isomerase, which in turn affects glycolysis in tumor cells [92].

LncRNA UCA1 plays an important role in bladder cancer via the activation of PI3K/AKT/mTOR pathway. Li et al. discovered that UCA1 can stimulate glycolysis by upregulating HK2. Earlier research has shown that STAT3 is a direct transcriptional activator of HK2. It is also a downstream effector of mTOR [93–95]. The authors further validated the association of UCA1 with the mTOR-STAT3 signaling pathway. The results showed that both rapamycin and STAT3 siRNA could decrease glucose consumption and lactate production, indicating that UCA1 can induce the expression of HK2 via mTOR-STAT3 pathway, thus regulating glycolysis [96] (Fig. 3).

### p53 signaling pathway

The absence of p53 in the cell can lead to mitochondrial respiratory damage and increased glycolysis [97, 98]. p53 not only inhibits the expression of GLUT1 and GLUT4 [99], but, it also acts as a transcription factor that regulates multiple metabolism-related enzymes [100]. Activation of p53 increases the ubiquitination of phosphoglycerate mutase (PGM), preventing the conversion of fructose-1,6-bisphosphate to pyruvate [101]. In gastric cancer, p53 inhibits glycolysis by activating TP53-induced glycolysis and apoptosis regulator (*TIGAR*) [102]. *TIGAR* is a p53-induced gene that encodes a protein, which degrades fructose 2,6-bisphosphate, which in turn prevents the activation of 6-phosphofructokinase 1 (PFK1), thereby inhibiting glycolysis. Therefore, glucose gets shunted into pentose phosphate pathway, which produces more NADPH. NADPH can produce a simplified form of glutathione, which is the main substance that protects the cell from ROS damage [103]. In conclusion, the multifaceted role of p53 in glucose metabolism in cancer is manifested in the inhibition of glycolysis and facilitation of TCA cycle and oxidative phosphorylation.

Wu et al. showed that a double mutant of p53 (N340Q/L344R) could facilitate the progression of HCC by upregulating PKM2. The p53 mutant forms a complex with LncRNA CUDR. The complex binds to the promoter regions of PKM2, enhancing the phosphorylation of PKM2 and its polymer formation [104]. Many lncRNAs can regulate the expression of p53 directly or indirectly. Maternally expressed gene 3 (MEG3) is usually absent in a variety of human tumor cell lines. MEG3 overexpression leads to an increase in p53 protein and activation of p53 downstream target genes [105]. MEG3 promotes p53-regulating transactivation in meningioma cell lines [106]. Wrap53, a natural antisense transcript of p53, regulates the mRNA level of endogenous p53 and induces its expression by targeting the 5′ UTR [107]. LincRNA p21 is a downstream transcript of p53. It can inhibit the transcription of p53 and induce apoptosis by binding to hnRNP-K [59]. MALAT1 is highly expressed in lung cancer, pancreatic cancer, non-small cell lung cancer, and is closely associated with cancer metastasis in patients with non-small cell lung cancer. Tripathi et al. found that knocking out MALAT1 in normal human fibroblasts stimulated DNA damage repair and resulted in the activation of p53 and its downstream target genes. The cell cycle defects observed in MALAT1-depleted cells were sensitive to p53 levels, indicating that MALAT1 may be an important inhibitor of p53 [108]. ROR is a special lncRNA in p53 signaling pathway. It can inhibit p53 and in turn be regulated by p53 [59]. These results suggest that lncRNAs may play a crucial role in p53-mediated regulation of glucose metabolism (Fig. 3).

## Therapeutic potential of lncRNAs in targeted treatment of cancer

Targeted therapy has attracted significant attention in recent times. Detailed understanding of lncRNA-mediated regulation of glucose metabolism in tumor cells may facilitate the development lncRNA inhibitors, which block tumor progression. Anti-miRNAs have been developed for treating hepatocellular carcinoma and are now in clinical trials [109]. Understanding the role of lncRNA in regulating glucose metabolism in cancer is important to explore the possibility of using lncRNA for targeted therapy.

In a recent study of lung adenocarcinoma, reversing the Warburg effect by inhibiting the EDFR signal pathway inhibited tumor development [65]. Pusapati et al. identified the mTORC1-dependent reprogramming of metabolism that allowed cancer cells escape dependence on glycolysis. Using a combination of targeted glycolysis and mTOR inhibitors to prevent metabolic reprogramming induced cancer cell apoptosis [110]. In the MCF-7 breast cancer cells, combination treatment using acarindine (AICAR) and Methotrexate (aminoglucuric acid) reversed the Warburg effect. Mono drug therapy may induce drug resistance, but combination therapy can induce the expression of AMPK and FOX1, resulting in increased mitochondrial oxidative phosphorylation and decreased glycolysis. These metabolic changes suggest an anti-Warburg effect that blocked the G1/S and the G2/M transition, slowing down cell cycle [111]. These results highlight the potential of targeting glucose metabolism for cancer treatment.

Fluorodeoxyglucose positron emission tomography (FDG-PET) has been employed to measure glucose metabolism, for detecting cancer and predicting the prognosis [112]. Current methods, including positron emission tomography (PET), autoradiography and magnetic resonance imaging (MRI), can measure the rate of primary metabolism of glucose. The limitations of these methods include their inability to distinguish markers and intermediate products. Germline mutations in succinate dehydrogenase and fumarate hydratase of TCA cycle have been reported in kidney and ganglion cell tumors [113]. One of the effects of these mutations is the activation of HIF-1α-regulated glucose metabolism. HIF plays a pivotal role in tumor metabolism; but HIF also regulates a variety of target genes, such as those involved in cell proliferation, angiogenesis and glucose metabolism. Therefore, therapies targeting HIF may cause unpredictable pathophysiological changes. Hence, it seems more reasonable to develop specific inhibitors targeting lncRNA.

In contrast to gene therapy, oligonucleotide therapy is more similar to small molecule therapy. Oligonucleotides can be synthesized artificially, do not integrate into the host genome and are highly specific. Therefore, they have minimal non-specific and generalized effects. Oligonucleotide-based therapies include using siRAN, anti-miRs, miRNA mimics, antisense oligonucleotides, targeting the upregulation of mRNA by lncRNA, and oligonucleotide-induced differential splicing [114]. Locked nucleic acid gapmers can effectively interfere with lncRNA. Gapmers have been reported to be effective in targeting primate PSCK9, but failed in phase 1 clinical trials [115]. Survivin and HIF-1α gapmers have been used for one year without safety issues [116, 117]. LncRNA-based targeted therapies still have a long way to go. Future studies need to address these exciting hypotheses.

## Prospect

Reprogramming glucose metabolism is a recently identified hallmark of cancer cells. Mounting evidence shows that numerous factors are involved in this process. We have highlighted the special roles of lncRNAs in this review. As discussed above, the interaction of lncRNAs with crucial transcription factors or metabolic enzymes involved in the processes of glycolysis can effectively modulate glucose metabolism and promote tumor progression. In addition to these critical molecules, other metabolic pathways are also pivotal for glucose metabolism in cancer, especially the PI3K/AKT/mTOR pathway and the AMPK pathway. LncRNA, as a regulator of metabolism, may provide novel attractive targets for cancer therapy. Therefore, detailed understanding of the role of lncRNA in regulating glucose metabolism and the mechanism by which it accomplishes this regulation will help to develop novel means to control aberrant metabolic phenotype and find more effective therapeutic strategies to suppress the “Warburg effect”, ultimately paving the way for better treatment of cancer.

## Abbreviations

ACC: Acetyl coA carboxylase
ACL: ATP-citrate lysase
CREB/CRTC: Cyclic AMP-responsive element -bingding protein/CREB-regulated transcription coactivator
CRNDE: Colorectalneoplasia differentially expressed
EGFR: Epidermal growth factor receptor
EMT: Epithelial-mesenchymal transition
F6P: Fructose-6-phosphate
FBP: Fructose bisphosphate
FDG-PET: Fluorodeo-xygucose positron emission tomography
G6Pase: 6-phosphoglucanase
GLUT: Glucose transporter
HIF: Hypoxia-induced factor
HK2: Hexokinase2
HOTAIR: Hox transcript antisense RNA
LDH: Lactic dehydrogenase
LINK-A: Long intergenic non-coding RNA for kinase activation
LKB1: The liver kinase B1
lncRNA MALAT1: Metastasis-associated lung adenocarcinoma transcript 1
lncRNA NBR2: Neighbour of BRCA1 gene 2
LncRNA: Long non-coding RNA
MEG3: Maternally expressed gene 3
mTOR: Mamalian target of rapamycin
OXPHOS: Oxidative phosphorylation
PDH: Pyruvate dehydrogenase
PDK1: Pyruvate dehydrogenase 1
PEPCK: Phosphoenolpyruvate carboxykinase
PFK: Phosphoric acid fructose kinase
PFK1: 6-phosphofructokinase 1
PGM: Phosphglycerate mutase
PI3K: the phosphoinositide 3-kinase
PK: Pyruvate kinase
PKM2: Pyruvate kinase 2
PML: Promyelocytic leukemia
PTENpg1: PTENpseudogene
ROS: Reactive oxygen species
STAT: Signal transducer and activator of transcription
TIGAR: TP53-induced glycolysis andapoptosis regulator
VHL: Von Hippel-Lindau
